# The Effect of Edible Chitosan Coatings Incorporated with *Thymus capitatus* Essential Oil on the Shelf-Life of Strawberry (*Fragaria x ananassa*) during Cold Storage

**DOI:** 10.3390/biom8040155

**Published:** 2018-11-21

**Authors:** Keydis Martínez, Marta Ortiz, Alberto Albis, Clara Gilma Gutiérrez Castañeda, Mayra Eliana Valencia, Carlos David Grande Tovar

**Affiliations:** 1 Programa de Ingeniería Química, Facultad de Ingeniería, Universidad del Atlántico, Carrera 30 Número 8-49, Puerto Colombia 081008, Colombia; keydismartinez@mail.uniatlantico.edu.co (K.M.); mrortiz@mail.uniatlantico.edu.co (M.O.); albertoalbis@uniatlantico.edu.co (A.A.); 2 Programa de Microbiología, Facultad de Ciencias Exactas y Naturales, Universidad Libre seccional Barranquilla, km. 7 vía a, Puerto Colombia 081008, Colombia; clarag.gutierrezc@unilibre.edu.co; 3 Escuela de Ingeniería de Materiales, Facultad de Ingeniería, Universidad del Valle, Calle 13 Número 100-00, Santiago de Cali 760032, Colombia; valencia.mayra@correounivalle.edu.co; 4 Programa de Química, Facultad de Ciencias, Universidad del Atlántico, Carrera 30 Número 8-49, Puerto Colombia 081008, Colombia

**Keywords:** chitosan, edible coating, essential oils, shelf-life, strawberry

## Abstract

The strawberry is a fruit appreciated in the food industry for its high content of bioactive compounds. However, it is considered a highly perishable fruit, generally attacked by pests of phytopathogenic origin, which decreases its shelf-life. Normally, to diminish the losses caused by pathogenic microbes, coatings of polysaccharides in combination with natural products like essential oils are applied. In this work, we describe the effect of edible coatings from chitosan (CT) incorporating *Thymus capitatus* essential oil (TCEO), applied to strawberries stored under refrigeration conditions (5 ± 0.5 °C). Different concentrations of TCEO were applied to chitosan coatings, with different effects on the physical and microbiological properties of the strawberries. All the products had greater acceptance and quality than the controls, being more effective those with essential oil incorporation. It is noteworthy that all the essential oil treatments lead to an increase in the shelf-life of strawberries of up to 15 days. Scanning electron microscopy (SEM) analysis of the microstructure showed a decrease in compactness with TCEO introduction, but without compromising food preservation after 15 days. In addition, treated strawberries delayed the loss of physicochemical and antioxidant properties, due to protection against the microbial development of aerobic mesophylls, molds, and yeasts.

## 1. Introduction

The strawberry (*Fragaria x ananassa*) is a nutraceutical fruit, appreciated in the food industry for its high content of bioactive compounds, such as vitamin C and phenolic constituents with antioxidant capacity [1]. Usually, it is consumed fresh or processed in juices.

In Colombia, losses of food from agriculture correspond to 64% of the total produced, mainly during the stages of production, post-harvest, storage, and industrial processing [2]. Among these foods, the strawberry is considered a highly perishable fruit, generally attacked by pests of phytopathogenic origin, which decreases its shelf-life and consumer acceptance [3].

Fruits and vegetables are rich in water and nutrients, and, during storage, are ideal substrates for development of pathogenic microorganisms, such as *Penicillium* spp., *Monilinia* spp., and *Botrytis* spp. In addition to the resulting economic losses, some pathogenic postharvest fungi, such as *Aspergillus* spp. and *Penicillium* spp., represent a serious human health concern due to the toxins produced (ochratoxin A, patulin, citrinin, etc.) [4,5]. Hence, the use of appropriate technologies is important to reduce losses, which increase during the maturation and storage of fruits [6].

Fungicide remains one of the most effective treatments to reduce postharvest decay, since it prevents the fungi infection before, during, and after marketing. However, repeated and continued use of fungicide has led to the development of strains with resistance to common fungicides, such as benzimidazole, dimethyl inhibitors, and dicarboximide [7].

The most common physical treatments are heat application (via hot water) above 40 °C for short periods (e.g., 55–60 °C for 20–60 s). However, excessive heating can produce weight loss, discoloration, and flavor changes [8,9]. 

An alternate physical treatment method is irradiation, in which the produce is exposed to a radiation for a short period of time, and which is considered a safe control method to extend the shelf-life of fruit [10]. The most common irradiation technology applied is gamma irradiation [11]. However, depending on the dose of irradiation applied, changes in product quality have been observed. 

Another technology employed to reduce the postharvest decay of fruit is the use of the generally recognized as safe (GRAS) chemicals by the US Food and Drug Administration (FDA): peracetic acid, K-sorb, sodium bicarbonate, and calcium salts. However, despite good results at the lab-scale level, these chemicals lack large-scale application, since they have low persistence and inconsistent activity [7]. Another treatment to reduce the postharvest decay of fruits is the application of electrolyzed water using sodium chloride as an electrolyte, to generate chlorine in situ as a sanitizer. The production of organic chlorinated compounds, such as chloramine, dichloramine, and trichloromethanes, is also reported, together with several drawbacks and concerns from consumers [12].

Because of the short-comings of the methods presented above, another viable alternative is the use of natural compounds, such as volatile organic compounds and essential oils [13,14], which are natural secondary metabolites produced by plants and are generally recognized as safe for applications in fruits. However, due to their high volatility they must be included within a matrix with lower volatility, such as that of polysaccharides, which reinforces antimicrobial and barrier properties. One example is chitosan, which has been reported as usefully extending the shelf-life of fruit [15].

Several preservation techniques, such as refrigeration, synthetic chemical fungicides, modified atmosphere packaging [16,17], osmotic treatments [18], hypobaric treatments [19], heat treatments [9], and edible coatings [20], have been used to study the effect on the postharvest decay of strawberries. Research has also encompassed edible coatings with semipermeable films through a reduction of moisture, gas exchange, respiration, and oxidative reaction rates [13,20,21].

Different studies have investigated the effect of edible coatings based on chitosan [20,22,23,24], chitosan combined with essential oils [3], chitosan-beeswax [25], chito-oligosaccharide [23], carboxymethyl cellulose, and hydroxypropyl methylcellulose [26] in strawberries, with interesting conclusions about their effects on postharvest quality. A study combining cold storage and chitosan treatments to increase the postharvest quality of fruit was also reported. The authors concluded that chitosan-coated fruit exhibited a slower rate of deterioration compared to uncoated fruit in all tested cultivars, based on the antioxidant enzymatic responses from strawberries [24].

The use of edible coatings is an alternative that has generated good results when used in different dietary matrices, particularly for fruits and vegetables, because they are a product of a clean technology, and have a high degree of selectivity and great economic feasibility. Among the coatings used, those based on polysaccharides, proteins, lipids, and mixtures thereof are of the greatest interest due to their properties. Chitosan (CT) is a polysaccharide [27,28] that has been widely studied as an edible coating due to its biocompatibility, biodegradability, and functionality, which make it versatile and highly attractive for various industries [29].

Due to their high antioxidant, antimicrobial, and antifungal properties, in recent years chitosan coatings have been used for their film-forming characteristics and their ability to act as gas and oxygen barriers, as well as to reduce loss of moisture and improve the respiration rate of the product [30]. Chitosan is considered an excellent defense against various factors that cause both biotic and abiotic deterioration [31].

Natural products, such as essential oils and other extracts of aromatic plants, have also recently been applied in edible films to improve physicochemical, antimicrobial, and antioxidant properties, as a potential alternative to traditional chemical contaminant preservatives [32].

The essential oils of the *Thymus* species are used for different applications, such as cosmetics, perfumery, and medicine [33]. With significant antioxidant and antibacterial properties, they can also be used by the food industry as possible natural additives to reduce the use of chemicals. Their effectiveness as antibacterial agents has been demonstrated in model systems that closely simulate the composition of foods [34]. Ballester-Costa et al. reported that the incorporation of essential oils of Thymus genera into chitosan films showed favorable results due to their antimicrobial properties, attributed mainly to the high content of bioactive compounds such as thymol and carvacrol [32,34,35].

*Thymus capitatus* (L.) *Hoffmanns* et Link (sin. *Corydothymus capitatus*), an aromatic plant from the Mediterranean region known as “Spanish Oregano”, is used as a food additive, although some medicinal applications have also been reported [36]. Different biological properties of *Thymus capitatus* (L.) *Hoffmanns* et Link have been reported, such as antibacterial, antifungal, antioxidant [37,38], hypoglycemic [39], and antiviral activities [40]. 

Our group reported the physical-chemical properties and antibacterial activity of chitosan coating incorporated with *Thymus capitatus* essential oil (TCEO) against the bacteria involved in the spoilage of tuna and swordfish [41]. A commercial TCEO was selected, as it contained reinforced main components and had a high degree of purity, which proved to be an advantage in increasing antimicrobial properties and in decreasing the fungal decay of fruits [41].

However, so far, TCEO has not yet been included in chitosan matrices to preserve the shelf-life of strawberries. Studying the effects of CT-TCEO coatings on the postharvest decay and antioxidant properties of strawberries could thus be highly promising. By combining the antimicrobial and barrier properties of chitosan with the antifungal properties of TCEO, research could yield a more effective treatment to extend the shelf-life of strawberries, which would benefit producers and the wider food industry.

## 2. Materials and Methods

### 2.1. Materials

#### 2.1.1. Fruit samples

Strawberries, collected from a local market, were selected, during the post-harvest stage, with uniform size, shape, and color, without any sign of mechanical damage or fungal deterioration. Subsequently, they were washed with 1% (*v*/*v*) sodium hypochlorite solution, before the coatings were applied, which included T1 = CT solution (containing chitosan in 2% *v*/*v* in a 1% acetic acid solution), T2 = chitosan solution + 0.5% of TCEO, T3 = CT + 1.0% of TCEO, T4 = CT + 1.5% TCEO, and T5 = uncoated control samples (strawberries washed with sodium hypochlorite 1%, sterilized water, and air-dried for one hour before storage), without any further treatment, to avoid false inhibition effects or affect color and physical properties, according to [42,43]. A total of 530 fruits were used to perform all the experiments, which were stored under refrigeration conditions at 5 °C ± 0.5 °C with a relative humidity of 70% in a refrigerator.

#### 2.1.2. Essential oil of *Thymus capitatus*

Essential oil of *Thymus capitatus* was acquired from Sigma, and its composition was determined by gas chromatography-mass spectrometry (GC-MS) according to the methodology reported by [44]. Briefly, the composition was determined on an AT 6890 Series plus gas chromatography spectrometer, with a mass selective detector (full scan). The analysis was carried out using a DB-5MS fused silica capillary column. The temperature program was 60 °C for 10 min, then heating at 5 °C/min to 250 °C, and maintained for 10 min. Other operating conditions were the following: helium (99.99%) as the carrier gas, with a flow rate of 1.1 mL/min; injection volume of 2:1 and split ratio 1:30; and on the other side, 0.1 μL of the samples were injected manually in split mode. The identification of the components was performed using electron ionization (EI, 70 eV), with a mass range from *m*/*z* 50–550. The constituents were identified by comparing their RI (retention index) with those provided in the literature and comparing the mass spectra with those recorded by Adams database (Wiley, 138 and NIST05, for Agilent, Santa Clara, California, USA).

### 2.2. Preparation of Edible Coatings

The antimicrobial coatings were prepared according to the procedure reported by [45], which consisted of adding a specific amount of chitosan (CT) (degree of deacetylation = 85%; Mw = 190.000–310.000 Da, Sigma Milwaukee, WI, USA) to a 1% (*v*/*v*) solution of acetic acid to obtain a final concentration of 2% (*w*/*v*), with constant agitation. Subsequently, glycerol (Sigma, Milwaukee, WI, USA) was added at a level of 0.75 mL/g of chitosan as a plasticizer and stirred for 30 min. Then, 0.2% (*v*/*v*, with respect to the volume of essential oil) of Tween 80 (Sigma, Milwaukee, WI, USA) was added as an emulsifier to the above solution. After one hour, TCEO (Sigma, Milwaukee, WI, USA) was added to the mixture, to reach the final concentration required (0.5%, 1.0%, and 1.5% *v*/*v*, with respect to the chitosan solution), and stirred using an IKA T25-Digital Ultraturrax (Staufen, Germany) at 7000 rpm until homogeneity of the dispersion was obtained. The CT-TCEO solution was left standing for 24 h until gas bubbles were not observed.

### 2.3. Physicochemical Analysis of CT-TCEO Dispersions

Particle size, apparent viscosity, and percentage of total solids of the dispersions of CT-TCEO were determined according to reported methods.

#### 2.3.1. Particle size

For the analysis of the particle size, an AIMSIZER 2011 laser diffractometer was used. Two drops of the sample were placed in a quartz cell with distilled water (1:10 mL), and the measurement was performed according to the International Organization for Standardization [46].

#### 2.3.2. Apparent viscosity measurements

Apparent viscosity measurements were run according to the “Gardner methodology” [47]. Standard tubes with different viscosities were compared with the sample. One test tube was filled with the dispersions. The sample and standard tubes (Cat. No. VG-9100) were placed in a holder. The support was turned over, and it was observed which of the standard tubes coincided better with the bubble rise-time of the test tube containing the sample.

#### 2.3.3. Total solid content

Approximately one gram of the sample was placed in a previously weighed aluminum disk and dried in an oven at 105 °C for one hour. The percentage of soluble solids was calculated according to Equation (1):

(1) $$\%S=\left(\frac{P_{s}-P_{d}}{P_{m}-P_{d}}\right)*100$$

in which %*S* is the percentage of non-volatile solids in the sample (*w*/*w*), *P_d_* is the weight of dry and clean aluminum disk (g), *P_m_* is the weight of the sample plus the aluminum disk (g), and *P_s_* is the weight of the dry sample plus the aluminum disc (g), in accordance with [48].

#### 2.3.4. Scanning Electron Microscopy (SEM) analysis of cross-sectional films

To test the cross-sectional area of the films, they were fractured by immersion in liquid nitrogen and then placed on the sample holder and surface coated with a layer of gold on Denton Vacuum Model Desk IV equipment, to produce a conductive surface. Subsequently, the JEOL Model JSM 6490 LV microscope was used in secondary electron mode by an acceleration voltage of 20 KV. The micrographs of the cross-section were analyzed by SEM at 1000 × and 150× magnification, respectively. 

### 2.4. Application of Edible Coatings to the Strawberries

The strawberries were submerged in each of the treatments for 120 s, corresponding to T1 = CT, T2 = CT + 0.5% TCEO, T3 = CT + 1.0% of TCEO, T4 = CT + 1.5% TCEO, and T5 = control. Subsequently, they were air-dried for one hour at 23 °C. After the coating process was completed, the strawberries were packed in polyethylene terephthalate boxes (15 fruits/box) and refrigerated at 5 °C ± 0.5 °C for 15 days [43].

A full factorial design with two manipulated variables, type of treatment and time of storage, was employed in this work. Five treatments were employed in all the experiments, but the number of levels of the time of storage was different for each response variable: for physicochemical variables (pH, total soluble solids, acidity, maturation index, weight loss, and decay index) there were eight levels, except for respiration rate, for which four levels were studied; for microbiological variables (count of aerobic mesophylls, and molds and yeasts) there were four levels; for antioxidant activity, four levels; and for sensorial analysis, two time levels. All tests were run in duplicate.

### 2.5. Physicochemical Measurements of Strawberries

For the physicochemical analysis of soluble solids, pH, titratable acidity, and maturation index, 150 strawberries were used on days 0, 1, 3, 6, 9, 12, 14, and 15, after coating treatments, following the parameters established by the standard NTC 4103 [49]. 

#### 2.5.1. pH and total soluble solids (TSS)

The pH was measured with a Thermo-Fisher Scientific pH meter, previously calibrated with buffer solutions of pH 4, 7, and 10. The samples implemented for the five treatments consisted of 5 g of the fruits macerated and blended in an Oster Mod. 4655 and homogenized with 50 mL of distilled water (NTC 4103 [49]). Soluble solids were measured with a Milwaukee MA871 refractometer at 23 °C.

#### 2.5.2. Titratable acidity

Five grams of previously treated fruits was mixed with 50 mL of distilled water and centrifuged. The mixture was titrated with 0.1N NaOH (NTC 4103, fresh fruit, Chandler strawberry) using phenolphthalein as indicator. The results were expressed as the percentage of citric acid [50], according to Equation (2).

(2) $$\%\;Citric\;acid=\frac{V_{1}\times N}{V_{2}}\times K\times 100$$

in which *V*_1_ is the volume of NaOH consumed (mL), *V*_2_ is the sample volume (mL), *K* is the equivalent-weight of citric acid (0.064 g/meq), and N is the normality of NaOH (0.1 meq/mL).

#### 2.5.3. Maturation index

This was calculated as the quotient of soluble solids and acidity (Equation (3)) [51].

(3) $$MI=\frac{{}^{\circ}BRIX}{ACID}$$

#### 2.5.4. Weight loss

The weight loss was determined by gravimetric analysis, using Equation (4) [43]. For this test, 150 strawberries were used and evaluated on days 0, 3, 6, 9, 12, and 15.

(4) $$\%PP=\frac{\left(Pi-Pf\right)}{Pi}\times 100$$

in which *%PP* is the percentage of weight loss and *P_i_* and *P_f_* are the initial and final weight of the sample (g), respectively. 

#### 2.5.5. Decay index

The methodology used to determine the fungal decay was reported elsewhere [52] and is briefly described here. The severity of fruit decay was evaluated visually according to the following scale: 1 = not damaged, 2 = light damage (<25%), 3 = moderate damage (>25% and <50%), 4 = severe damage (>50 and <75%), and 5 = completely damaged (75–100%) [53]. The decay index was calculated using the following formula [54]:

(5) $$Decay\;index=\frac{\sum \left(damage\;level\right)\left(N{}^{\circ}\;of\;fruits\;at\;this\;level\right)}{N{}^{\circ}\;of\;total\;fruits}$$

For this assay, 150 strawberries were used and evaluated on days 0, 3, 7, 11, and 15.

#### 2.5.6. CO_2_ production rate

For this assay, a closed system EcoChamber ME-6667 (PASCO, Roseville, California, United States) equipped with carbon dioxide sensor PS-2110 was used to measure the respiration rate. This was done in a 0.655 L hermetically sealed glass jar, equipped with a septum on the lid to take gas samples in the upper space, at different times for 10 h, every 30 min, using a needle connected to the gaseous CO_2_ analyzer on days 0, 2, 6, and 8, for each treatment.

### 2.6. Microbiological Quality

These analyzes were carried out in duplicate, for the count of aerobic mesophylls, molds, and yeasts, on days 0, 5, 10, and 15 of the treatments. During these days, strawberries of approximately 10 g of mass were taken; subsequently, they were homogenized for 10 min in 90 mL of peptonized water. The homogenate was serially diluted from 10–1 to 10–6, and after that 1 mL of each solution was poured into plates on the surface of the medium containing Potato Dextrose Agar (PDA) with 10% tartaric acid (p/v) for the test of molds and yeasts, and Plate Count Agar (PCA) for aerobic mesophyll, and was grown for a period of 3 days.

The mesophylls aerobic count was performed in accordance with ISO 4833 using the plate colony counting technique at 30 °C. The results were reported as logarithm colony-forming units per gram (Log CFU/g) of mesophylls bacteria. Mold and yeast counts were performed in accordance with ISO 7954, based on the plate colony counting technique at 25 °C. The results were reported as log UFC/g of molds and yeasts.

### 2.7. Antioxidant Activity

To evaluate the evolution of the antioxidant activity of the strawberry treatments during a period of time of 15 days, the methods of 2,2-diphenyl-1-picrylhydrazyl (DPPH) (during 30 min of reaction) and 2,2′-azino-bis (3-ethylbenzothiazoline-6-sulfonic acid) (ABTS) (during 7 min of reaction) were evaluated on days 0, 5, 10, and 15 of cold storage. These methods analyze the ability to trap radicals after treatment with coatings applied to strawberries (0%, 0.5%, 1.0%, and 1.5%). For the preparation of strawberry extracts, samples of 2.5 mg in 10 mL of methanol were homogenized. The homogenate was placed in an ultrasonic bath for 30 min at 4 °C and centrifuged. The supernatant was filtered on Whatman paper No. 1 [6]. With the obtained extract, the samples were prepared, in which 5, 10, 20, 40, and 70 μL of the solution were taken and mixed with absolute methanol until 1 mL was completed.

### 2.8. Sensorial Analysis

The test was carried out in the nutrition and dietetics laboratory of the Universidad del Atlántico, with the participation of 30 untrained panelists, considering the Colombian Technical Standard 3932 [55]. Two groups of 15 judges were formed, which performed the test independently during days 4 and 7 of the application of the coatings on the product, with a prior instruction.

### 2.9. Statistical Analysis

The analysis of variance (ANOVA) and the LSD method (least significant difference) for mean separation, with a confidence level of 95% (α = 0.05), were used to evaluate the effect of edible coatings in the response variables described above. The Statgraphics Centurion XVI program was used for these statistical analyzes.

## 3. Results and Discussion

### 3.1. Essential Oil Characterization

During the GC-MS analysis of the essential oil, 25 compounds were identified (Table 1). The compounds corresponded to 23 monoterpenes (99.7%), a sesquiterpene (0.2%), and an unidentified compound corresponding to the remaining percentage. In several papers, it has been reported that the monoterpenes exert antimicrobial, antiviral [56], and antifungal activity [57]. However, a sesquiterpene was also present in the essential oil and could be responsible for the biological activities. In addition, some investigations have been published on essential oils that provide sesquiterpenes as their main components [58], with interesting antimicrobial activity against spoilage bacteria from fishes.

### 3.2. Physicochemical Characterization of Chitosan Emulsions

The percentage of total solids (Table 2) accounts for the fraction of the sample that does not evaporate at 105 °C, which is mainly constituted by the polymeric matrix and essential oil. The coatings that presented the highest total solid contents were those with 1.5% of TCEO, as expected, since a 2% solution of chitosan and 1.5% of essential oil should account for 3.5% of theoretical solids, which corresponds with the value measured (3.27%), suggesting that most of the oil components were retained in the polymer matrix during the analysis.

The analysis of the particle size offers a parameter related to the stability of the emulsion, since a smaller particle size implies greater stability, because it increases the barrier properties of the coatings against moisture and gases [59]; therefore, it is expected that all the solution of essential oil presents good stability based on the particle size and that no separations were observed between the preparation and the time taken to use it for the coating experiments.

On the other hand, the incorporation of the essential oil had a substantial effect on the apparent viscosity of the emulsions. The apparent viscosity decreases when the percentage of the essential oil increases. This behavior can be explained by considering two opposite effects: the increase in the concentration of the dispersed phase (oil) tends to increase the apparent viscosity of the system, while the adsorption of the polymer on the droplet surface induces a decrease of its effective thickening concentration in the aqueous phase [60]. Similar behavior was found in chitosan containing basil, thyme [50], bergamot, lemon, and tea tree essential oils [61].

### 3.3. Morphological Analysis of CT-TCEO Films

**Figure 1:**
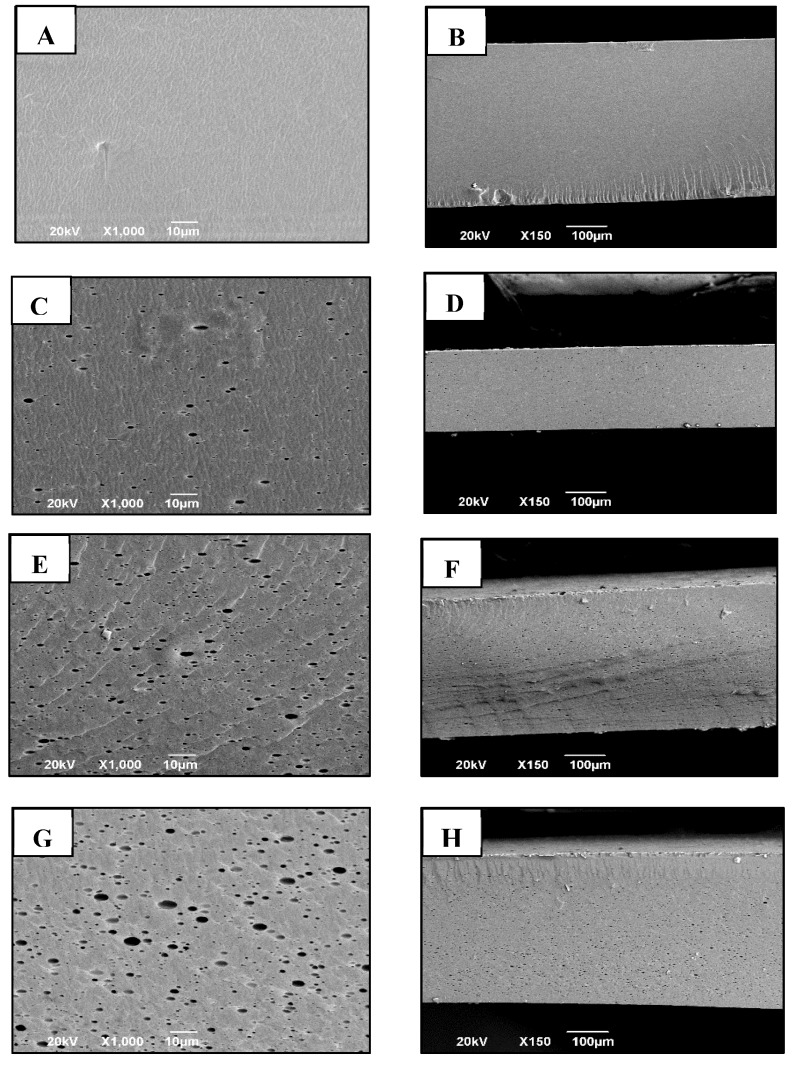
Cross-section analysis of CT-TCEO films with 0% (**A**) and (**B**), 0.5% (**C**) and (**D**), 1.0% (**E**) and (**F**), and 1.5% (**G**) and (**H**), at ×1000 and ×150 of magnification, respectively.

From the cross-section analysis of pure chitosan control films (Figure 1A,B), it can be observed that they are homogeneous, thin, and continuous, without porous defects or ruptures. The films incorporated with TCEO presented lower homogeneity in the structure, with the presence of porosity, in a sponge structure, presumably caused by the evaporation of the oil and accompanied by an increase in the roughness of the structure (C-H). With the increase of the essential oil content, an increase in the appearance of holes and defects in the microstructure was found, although the films continued to be resistant without loss of functionality. It is known that the loss of the structure of the films with incorporated TCEO is due to the essential oil components that interrupt the association of the chitosan chains between them, due to their hydrophobic nature, which is generated during the evaporation gaps between polymer chains. Similar results were observed in previous studies [42].

### 3.4. Physicochemical Analysis and Antimicrobial Properties of Coatings on Strawberries

#### 3.4.1. pH

**Figure 2:**
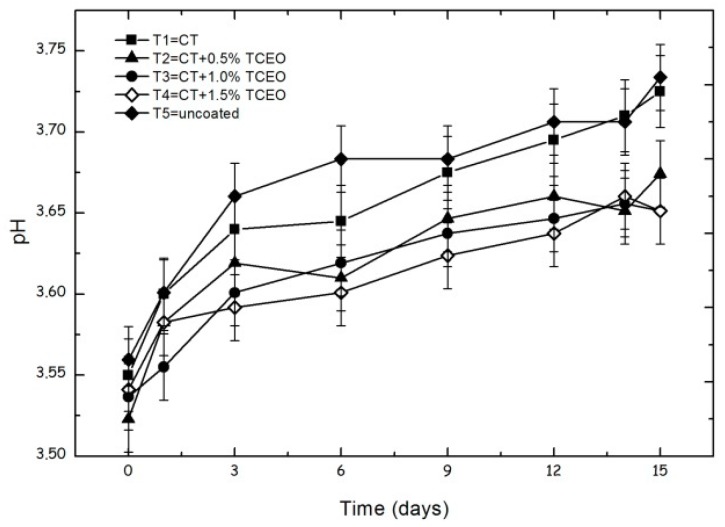
Evolution of pH in strawberries with chitosan (CT) and treatments of oil of Spanish Oregano (TCEO): T1 = CT, T2 = CT + 0.5% of TCEO, T3 = CT + 1.0% of TCEO, T4 = CT + 1.5% TCEO, and T5 = uncoated. Mean values and intervals of Fisher’s LSD of 95% according to the ANOVA test.

The pH of the solutions increased during the entire storage period (Figure 2). This behavior was attributed to the decrease in the amount of available organic acids, which are used as an energy source to sustain the fruit-ripening process [52].

Statistical analysis indicates significant (*p* < 0.05) changes in pH with treatments and storage time. Compared to the control, the pH of fruits coated with TCEO, [T2 (3.53 to 3.69), T3 (3.54 to 3.67), and T4 (3.55 to 3.67)] presented significant differences (*p* < 0.05). From Figure 2, it can be observed that treatments with TCEO delay the pH increment, and this effect is more pronounced as the TCEO content increases. The final result is the extension of the shelf-life of strawberries and the diminishment of the effect of the maturation of the fruit through the consumption of organic acids. 

#### 3.4.2. Total soluble solids

**Figure 3:**
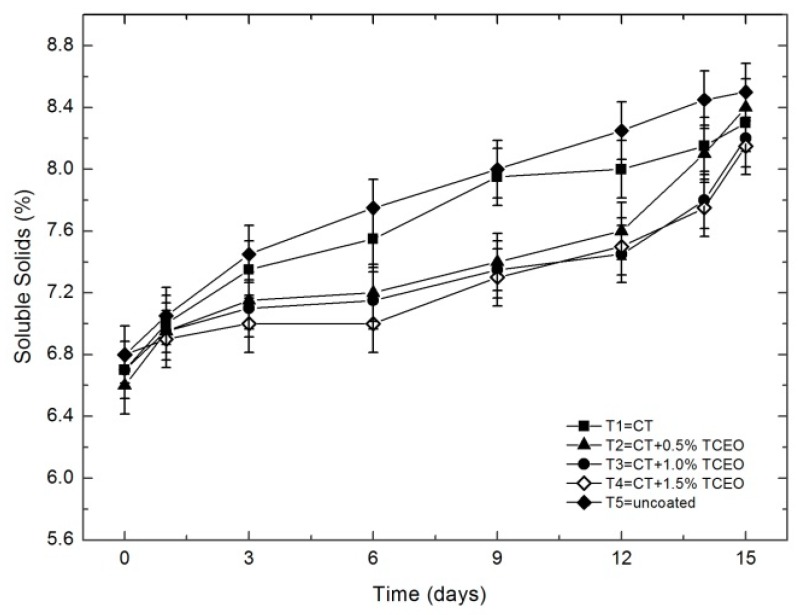
Evolution of the total soluble solids content by TSS measurement in strawberries with chitosan (CT) and treatments of oil (TCEO): T1 = CT, T2 = CT + 0.5% of TCEO, T3 = CT + 1.0% of TCEO, T4 = CT + 1.5% TCEO, and T5 = uncoated. Mean values and intervals of Fisher’s LSD of 95% according to the ANOVA test.

The statistical analysis indicates significant changes (*p* < 0.05) in the total content of soluble solids in the storage time (Figure 3). The control presented significant differences (*p* < 0.05) compared to the fruits covered with T2 (6.60 to 8.40), T3 (6.70 to 8.20), and T4 (6.80 to 8.15), which indicates that the coatings with *Thymus capitatus* prolonged the shelf-life of the strawberries by decreasing the metabolism and delaying maturation, as indicated by the results for the total content of soluble solids, which have been previously observed [62].

#### 3.4.3. Titratable acidity

**Figure 4:**
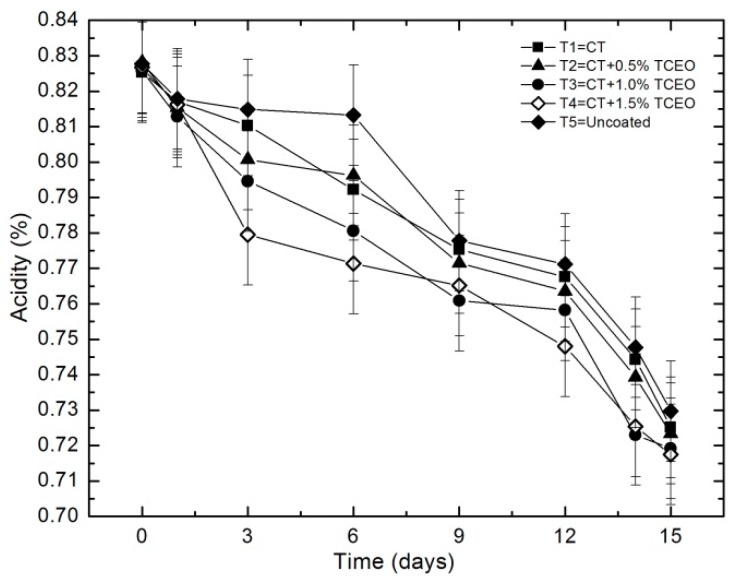
Evolution of the percentage of titratable acidity expressed as citric acid in strawberries with chitosan (CT) and treatments of oil (TCEO): T1 = CT, T2 = CT + 0.5% of TCEO, T3 = CT + 1.0% of TCEO, T4 = CT + 1.5% TCEO, and T5 = uncoated. Mean values and intervals of Fisher’s LSD of 95% according to the ANOVA test.

Figure 4 shows that acidity values decrease progressively as a function of storage time for all coated samples and control.

The decrease in this parameter (which is related to the increase in pH) is attributed to the sugar conversion, as a result of the enzymatic reactions during microbial respiration [26]. The statistical analysis indicated that there are significant differences (*p* < 0.05) in the titratable acidity of the fruits during the storage time, but not between treatments (*p* ≥ 0.05).

#### 3.4.4. Maturity index

**Figure 5:**
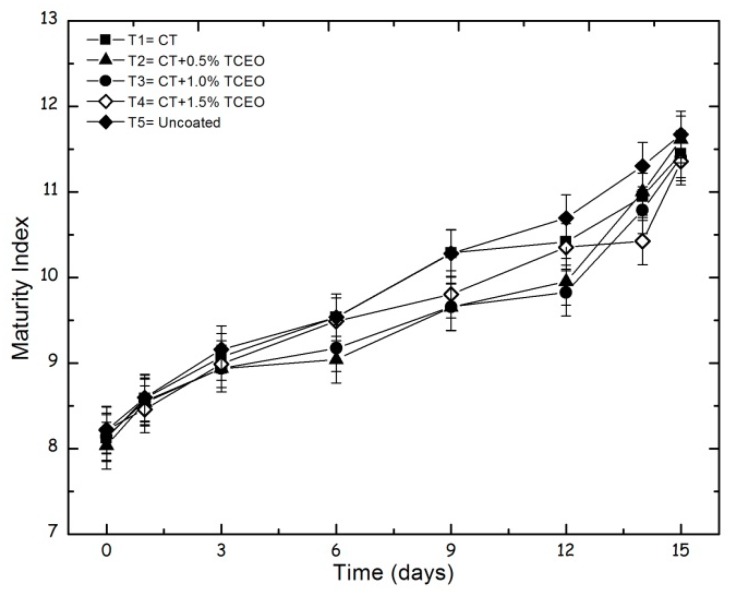
Evolution of the maturity index in strawberries with chitosan (CT) and treatments of oil TCEO: T1 = CT, T2 = CT + 0.5% of TCEO, T3 = CT + 1.0% of TCEO, T4 = CT + 1.5% TCEO, and T5 = uncoated. Mean values and intervals of Fisher’s LSD of 95% according to the ANOVA test.

Figure 5 shows that all treatments exhibit an increase in the maturation index over the storage time, in which the average values were 11.45 for T1, 11.62 for T2, 11.41 for T3, 11.36 for T4, and 11.67 for T5, during day 15 of storage.

The statistical analysis indicates significant changes (*p* < 0.05) in the maturity index in relation to the storage time, but not with the treatments (*p* ≥ 0.05). The addition of essential oil from the coatings of chitosan and with *Thymus capitatus* promoted a delay in the ripening of the fruits, probably due to the effect of the components of the essential oil on the metabolic activity of the fruit. Some authors argue that this type of compound within cell membranes can affect the metabolic pathway of the fruit and senescence, conserving the fruit for longer [61]. As previously reported, soluble solids content, pH, and maturity index increased throughout the storage time in agreement with the progress of the ripening process [52].

#### 3.4.5. Weight loss

**Figure 6:**
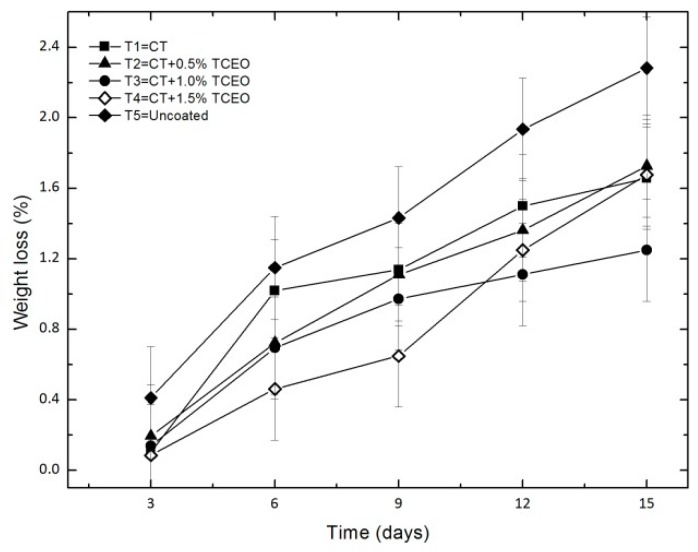
Evolution of weight loss percentage in strawberries with chitosan (CT) and treatments of oil (TCEO): T1 = CT, T2 = CT + 0.5% of TCEO, T3 = CT + 1.0% of TCEO, T4 = CT + 1.5% TCEO, and T5 = uncoated. Mean values and intervals of Fisher’s LSD of 95% according to the ANOVA test.

Figure 6 shows that all the samples experienced slight weight loss during storage time, although this was lower for the coated samples, due to the barrier effects exerted by the coatings that decreased the water loss of the samples. 

The statistical analysis shows significant differences (*p* < 0.05) in the weight loss during the storage time and with the treatments. The coating application of chitosan and CT-TCEO shows a reduction in the weight loss percentage. Notably, on day 15 the treatment that showed better behavior was T3, with a weight loss of 0.14%. Commonly, it is believed that the environment migration of water from the fruit to the environment is the main cause of the weight loss of the fruit. A careful observation of Figure 6 [63] demonstrated that until day 9, the samples that lost less weight were those that included more essential oil (only 1.5%, since 1.0%, and 0.5% remain almost in the same value). However, after that day, treatments with 1.0% and 1.5% seemed to lose more weight, presumably due to the excess of the essential oil that could volatilize after longer periods of time, when the strawberries texture and molecular structure could change, due to the high volatility of the TCEO. This fact has also been observed before by [52], where they analyzed the loss in the antifungal activity of some essential oils as a result of the high volatility of them.

#### 3.4.6. Decay index

**Figure 7:**
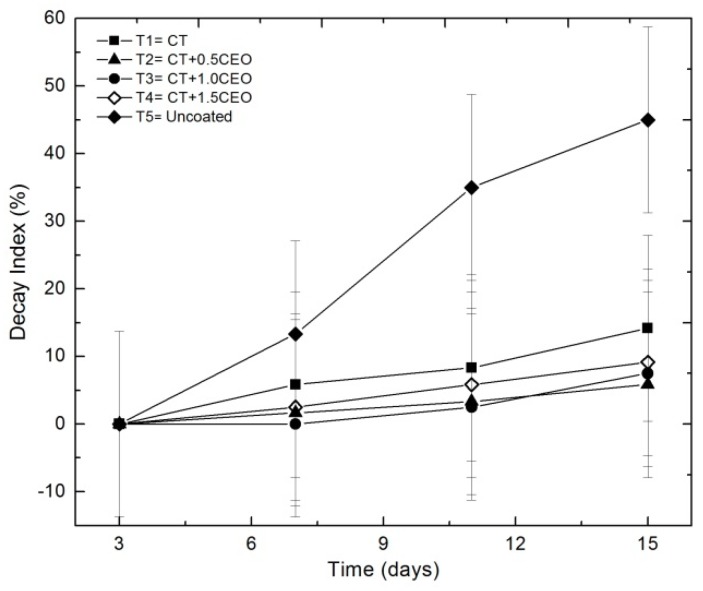
Evolution of the decay index in strawberries with chitosan (CT) and treatments of oil (TCEO): T1 = CT, T2 = CT + 0.5% of TCEO, T3 = CT + 1.0% of TCEO, T4 = CT + 1.5% TCEO, and T5 = uncoated under cold storage. Mean values and intervals of Fisher’s LSD of 95% according to the ANOVA test. Data were transformed using the logarithmic function prior to analysis.

The percentage of fungal decay increased during storage time for all treatments, as shown in Figure 7. The fungal decay during 15 days was 14.17% for T1, 5.83% for T2, 7.50% for T3, 9.17% for T4, and 45.0% for T5, respectively.

Decay index in strawberry fruit increased during storage time for all treatments, as shown in Figure 7. The statistical analysis indicates significant changes (*p* < 0.05) in the percentage of decay during storage time and treatments. The decay index in coating fruits after 15 days was lower than that in control: 14.17% for T1, 5.83% for T2, 7.50% for T3, 9.17% for T4, and 45.0% for T5, respectively. The coatings were more efficient at preserving the quality of strawberries for 15 days, since the decomposition decreased in comparison with the untreated samples (*p* < 0.05). It should be noticed that the treatments with TCEO were the most efficient at reducing the fungal decomposition (35% to 39%) and could be considered an effective antifungal of the species that causes the deterioration of the strawberries in refrigeration. The growth of fungi on the surface of the strawberries under refrigeration conditions decreased the number of purchases by consumers, and the edible coatings used here allowed one to increase the shelf life in refrigeration conditions. This result is in accordance with expectations, considering that one of the functions of edible coatings is to act as a barrier between the fruit and the environment, which retards external microbial growth.

Different studies have demonstrated that fungi membrane mainly containing polyunsaturated free fatty acids are sensitive to chitosan activity [58,64].

Chitosan-essential oil coatings provide a semipermeable film surface that can reduce moisture transfer, oxygen uptake, respiration rate, and ethylene production, and carry additional functional ingredients (antioxidants or antimicrobial agents) that retard microbial growth [65].

Chitosan-essential oil coatings also inhibit spore germination, germ-tube elongation, radial growth, chelates metals, minerals, and trace elements or nutrients essential for the fungal growth [66], and can also affect fungal cell wall morphogenesis [67], which is mainly dependent on the chemical structure of Eos components and on their ability to penetrate the fatty acid chains of the lipid bilayer, rendering the cell membrane more permeable [68] and, finally, resulting in cell death or inhibiting the sporulation and germination of spoilage fungi as a result of the synergistic process [69,70,71].

Many publications on essential oils incorporated in chitosan coatings or films strongly demonstrated a broad-spectrum inhibitory effect against pathogenic, food-related fungi and improved preservation of fruits regarding fungal decomposition. Although the mechanisms of how the coatings of chitosan and essential oils act as antifungals are still unclear, it is suggested that they can act synergistically to improve antifungal activity [58].

As previously observed, the obtained results suggest that CT could reduce the gas permeability in the fruit surface, thus affecting the gas exchange during fruit respiration, but with the introduction of the essential oil content, the barrier effect also increased. However, the introduction of *Thymus capitatus* in the coatings might be affecting the metabolic pattern of strawberries, effect observed also in the increasing of the maturation index, pH, and solid soluble content, affecting the respiration of the fruits [52]. Besides that, the micropores structure of coating film acts as a barrier to gas slowing down the respiration rate and controlling the fruit decay [53].

Different studies have been reported using coatings of chitosan and essential oils to control fungal decay and extend the shelf-life of fruits and vegetables. For example, to increase the shelf-life of strawberries during storage [3,52,72] the effect of the application of chitosan coatings including essential oils has been studied, presenting good results in the inhibition of the fungi development and fungal decay of strawberries caused mainly by *Botrytis cinerea*, known as grey mold rot, which is a major postharvest pathogen that causes considerable losses in a wide variety of harvested commodities such as fruits, vegetables, and ornamental crops. The infection is characterized by a latent phase that often results in an active decay lesion during the ripening process.

In another study, EO treatments on the radial mycelial growth of *Colletotrichum gloeosporioides* (the most common plant pathogen in the world, which causes the common postharvest disease called anthracnose, which affects fruit quality, marketability, and shelf-life) was studied by [73]. After 10 days of incubation, the edible coating showed a fungicidal effect *in vitro*.

The combination of chitosan and thyme essential oil has also been shown to improve the postharvest quality of avocado when nanostructured edible coatings based on chitosan (1% and 0.05%)-thyme essential oil (1–5%) nanoparticles (CSTEO-NPs) were used against *C. gloeosporioides* [74].

#### 3.4.7. CO_2_ respiration rate

**Figure 8:**
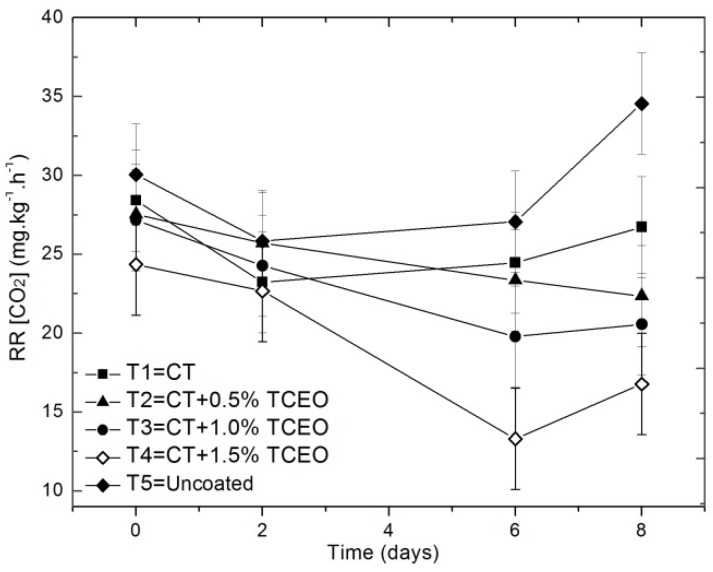
Evolution of respiration rate in strawberries with chitosan (CT) and treatments of oil (TCEO): T1 = CT, T2 = CT + 0.5% of TCEO, T3 = CT + 1.0% of TCEO, T4 = CT + 1.5% TCEO, and T5 = uncoated. Mean values and intervals of Fisher’s LSD of 95% according to the ANOVA test.

Strawberries are characterized by a high respiration rate, which is very dependent on temperature and storage time. Figure 8 represents the values of the respiration index, expressed as mg of CO_2_ kg^−1^h^−1^, on days 0, 2, 6, and 8. 

The statistical analysis shows that there are significant differences (*p* < 0.05) in relation to the respiration rate between fruits with treatments, but not with time (*p* ≥ 0.05). Among all the treatments designated, CT-TCEO 1.5% showed the best barrier properties against the evolution of CO_2_, which could be related to lower weight loss and lower fruit-decay, as previously shown. The results indicate that the use of essential oil in the coatings caused a progressive reduction of the respiration rate. This is similar to the chitosan treatments with lemon essential oil, which decreased the CO_2_ generation compared to the pure chitosan coating after 12 days of storage. As previously observed, the obtained results suggest that CT reduces the gas permeability in the fruit surface, thus affecting the gas exchange during fruit respiration, but with the introduction of the essential oil content, the barrier effect also increased. However, the introduction of *Thymus capitatus* in the coatings might be affecting the metabolic pattern of strawberries, an effect that is also observed in the increasing of the maturation index, pH, and solid soluble content, affecting the respiration of the fruits [42]. 

Coatings are typically applied over the fresh fruits and vegetable surfaces, for slowing down respiration, preventing the loss of food aroma and flavor compounds, and delaying the ripening process. However, the modification of the internal atmosphere by the use of coatings can develop ethanol and alcoholic flavors as a result of anaerobic fermentation associated with excessively high carbon dioxide or excessively low oxygen concentrations [75]. For that reason, it is necessary to control the ethanol production or alcoholic flavors in sensorial panels. In our case, no alcoholic flavors were perceived by the panel.

### 3.5. Microbial Quality

#### 3.5.1. Aerobic mesophylls

**Figure 9:**
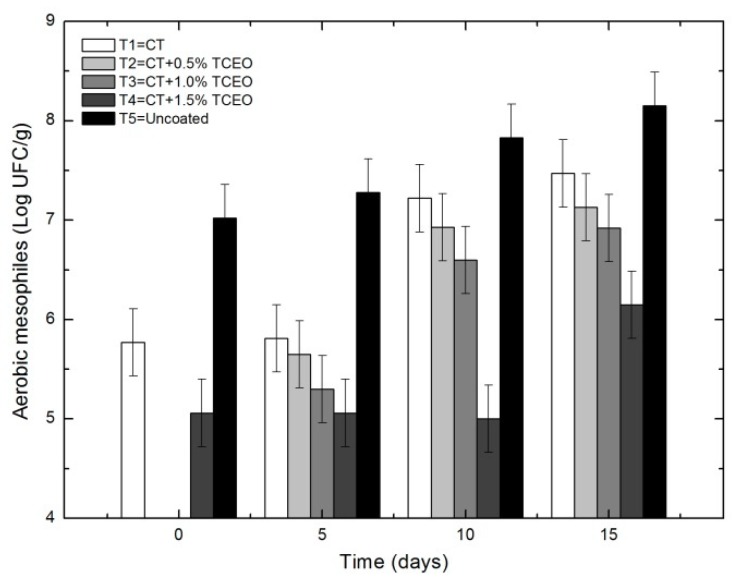
Effect of treatments on the concentration of aerobic mesophilic in strawberries with chitosan (CT) and treatments of oil (TCEO): T1 = CT, T2 = CT + 0.5% of TCEO, T3 = CT + 1.0% of TCEO, T4 = CT + 1.5% TCEO, and T5 = uncoated. Mean values and intervals of Fisher’s LSD of 95% according to the ANOVA test.

Figure 9 represents the values of aerobic mesophyll counts, expressed as Log UFC/g, during days 0, 5, 10, and 15. It can be seen that all the treatments showed lower growth of aerobic mesophylls compared to the control and were more effective those with CT-TCEO.

The statistical analysis indicated that there are significant differences (*p* < 0.05) in relation to the parameter of aerobic mesophylls in the storage time and between the treatments applied in strawberry. It can be observed that all coatings from chitosan (CT) incorporating essential oil of *Thymus capitatus* (TCEO) showed an increase in only one logarithmic unit of the total mesoaerobic population (*p* ≤ 0.05) compared to the control, in which these counts increased two logarithmic units. This could be the result of synergy generated by the combination of essential oil and chitosan, mainly due to the presence of terpenoid compounds that could have an antibacterial effect such as carvacrol, thymol, linalool, *p*-cymene, γ-terpinene, β-myrcene, and trans-β-caryophyllene. In general, chitosan by itself is capable of reducing microbial growth.

#### 3.5.2. Molds and yeasts

**Figure 10:**
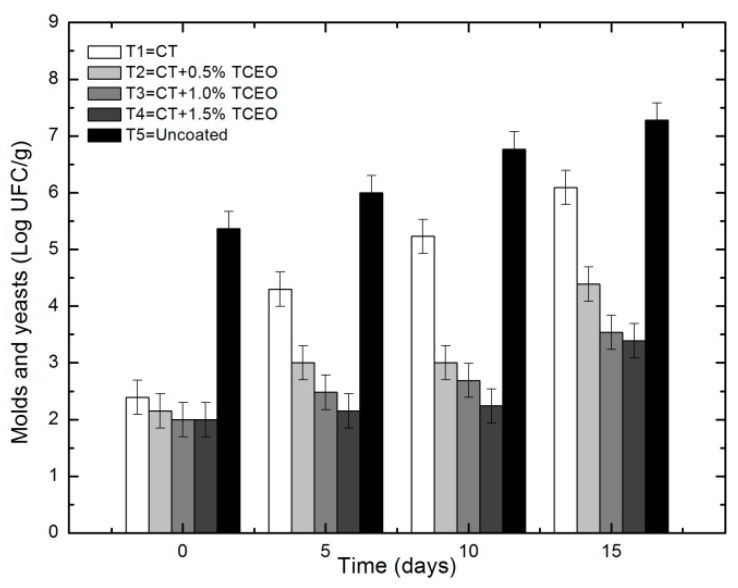
Counting of molds and yeasts in strawberries with chitosan (CT) and treatments of oil (TCEO): T1 = CT, T2 = CT + 0.5% of TCEO, T3 = CT + 1.0% of TCEO, T4 = CT + 1.5% TCEO, and T5 = uncoated. Mean values and intervals of Fisher’s LSD of 95% according to the ANOVA test.

Figure 10 shows the effect of edible coatings on the decrease in the growth of molds and yeasts in strawberries stored at 5 ± 0.5 °C.

The statistical analysis indicated that there are significant differences (*p* < 0.05) in relation to the parameter of molds and yeasts in the storage time and between the treatments applied to strawberries. With respect to the control, it can be observed that the treatments significantly reduced (*p* ≤ 0.05) the population of molds and yeasts, resulting in more effective the treatments with incorporation of *Thymus capitatus*, reaching a final population of T2: 4.39 Log CFU/g, T3: 3.54 Log CFU/g, and T4: 3.39 log CFU/g. The increase in the population of molds and yeasts between day 0 and 15 in the treatments with a coating of chitosan and essential oil of *Thymus* was of only one logarithmic unit; on the contrary, in the control treatment, this increase was of two logarithmic units. Different studies have shown that films and coatings based on chitosan and essential oils improved fruit quality and shelf-life due to the inhibitory properties of essential oils against pathogenic, food-related fungi [58]. In fruit-life studies, the aim is to maintain the population of molds and yeasts below values of 7 log CFU/g, as was achieved with the treatments used here throughout 15 days of testing. However, in the production and supply chain, many factors influence the concentration of the initial microbiota, which is why this methodology is useful if it is applied in the early stages of fruit harvesting.

### 3.6. Antioxidant Activity

#### 3.6.1. DPPH

**Figure 11:**
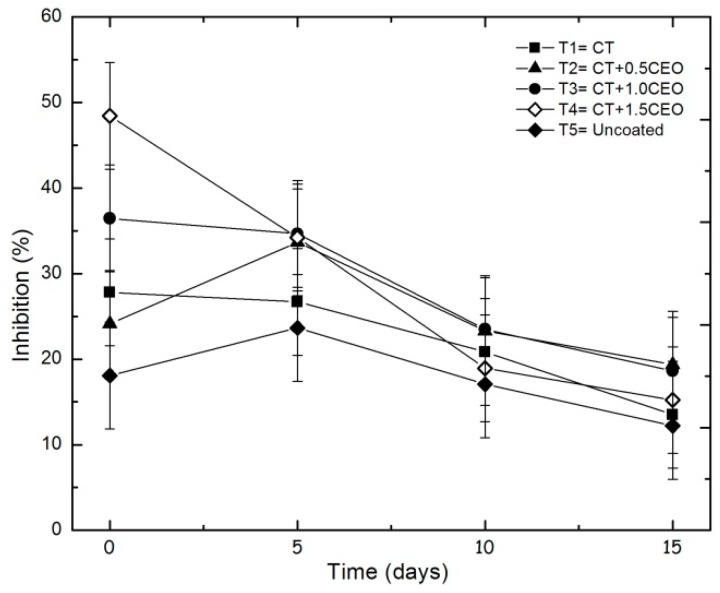
Evolution of antioxidant capacity by DPPH method in strawberries with chitosan (CT) and treatments of oil (TCEO): T1 = CT, T2 = CT + 0.5% of TCEO, T3 = CT + 1.0% of TCEO, T4 = CT + 1.5% TCEO, and T5 = uncoated throughout a 15-day period. Mean values and intervals of Fisher’s LSD of 95% according to the ANOVA test.

Figure 11 shows the evolution of the antioxidant activity with the DPPH technique throughout a 15-day period. The treatments with TCEO demonstrated that the effect to retain the antioxidant capacity is proportional to the essential-oil content, caused by the essential-oil components, especially those with phenol moieties [76].

The statistical analysis indicated that the antioxidant capacity by DPPH method presents significant differences (*p* < 0.05) during the storage time but not between treatments (*p* ≥ 0.05). The fruits with *Thymus capitatus* retained a higher antioxidant capacity, reinforcing this attribute in the fruit; the decreasing in this property at the end of the storage time could be due to senescence and decomposition [77].

#### 3.6.2. ABTS

**Figure 12:**
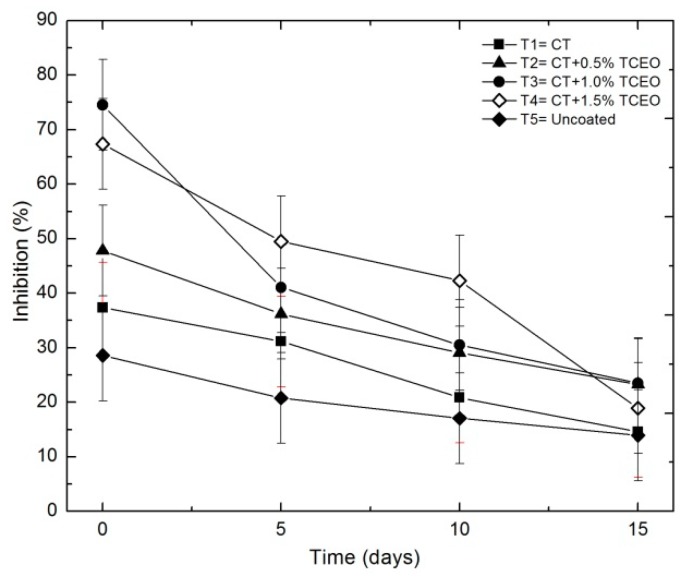
Evolution of the antioxidant capacity by the ABTS method in strawberries with chitosan (CT) and treatments of oil (TCEO): T1 = CT, T2 = CT + 0.5% of TCEO, T3 = CT + 1.0% of TCEO, T4 = CT + 1.5% TCEO, and T5 = uncoated throughout a 15-day period. Mean values and intervals of Fisher’s LSD of 95% according to the ANOVA test.

Figure 12 shows the evolution of the antioxidant activity of the strawberries, with each of the treatments. As can be observed, during day 15 of storage, the percentage of inhibition of the ABTS°+ radical was higher in the fruits with the incorporation of *Thymus capitatus* (T2, T3, and T4) compared to the control (T1). As shown in Figure 11, the percentage of inhibition reached values of 14.57, 23.29, 23.50, 18.93, and 13.93 for T1, T2, T3, T4, and T5, respectively, at the end of the test. 

The statistical analysis indicated significant differences (*p* < 0.05) in the antioxidant capacity by ABTS method during the storage time and between the treatments applied to strawberries (*p* ≥ 0.05). The addition of CT-TCEO delayed the reduction of the antioxidant capacity, resulting in a beneficial effect for the strawberries, retaining its antioxidant capacity under storage and refrigeration conditions.

The most commonly applied methods to study the antioxidant ability are ABTS and DPPH. Both have excellent stability under certain conditions, although they also show differences. DPPH is a free radical that can be obtained directly without previous preparation, while the ABTS has to be generated after a reaction that can be chemical, enzymatic, or electrochemical [78]. However, the results obtained with the methods allow one to reach practically similar conclusions. 

The oxidative degradation of lipids and some other molecules is one of the main factors limiting the shelf-life of food products. In recent years, several undesirable disorders have been detected as side-effects of using commonly used synthetic antioxidants. Using a multiple-method approach, the authors of [79] evaluated the antioxidant ability of oregano (*Origanum vulgare*), thyme (*Thymus vulgaris*), rosemary (*Rosmarinus officinalis*), sage (*Salvia officinalis*), and clove (*Syzygium aromaticum*) essential oils [79]. 

Essential oils are important natural antioxidant extracts that have gained significant attention in recent decades [80]. These properties are an inherent ability of some of their components, particularly phenols, to stop or delay the aerobic oxidation of organic matter, mainly due to the radical chemistry of some terpenoids and other volatile constituents [81]. For example, when molecules such as γ-terpinene are mixed with an unsaturated lipid in sufficient amount, they will reduce the overall rate of oxidation [81].

Usually, in ABTS and DPPH methods, colored persistent radicals are used as probes. They are reduced by the antioxidant, and the color change in the solution is measured by UV−vis spectrophotometry. However, these tests use probes that are chemically very different from the radicals responsible for the autoxidation of real systems. The result indicates a “radical trapping power” rather than true antioxidant activity [81]. 

All the limitations and solutions for these methods as indicators of antioxidant assays have been discussed before [81,82]. In general, short reaction times (less than five minutes) should be used to obtain results that reflect real radical scavenging power. However, several publications have reported the positive effect of edible films of chitosan incorporated with the EO of thymus exhibiting good antioxidant as well as antibacterial effects [83]. 

The main reason for the antioxidant activity observed for essential oils of the *Thymus* spp. has been attributed to the carvacrol and thymol content [84,85].

The antioxidant properties of the essential oil from thymus species in relation to its chemical composition have been evaluated recently, demonstrating that the antioxidant activity of essential oil is less effective than the ascorbic acid but comparable with the α-tocopherol and the synthetic antioxidant butylated hydroxytoluene (BHT), indicating a good antioxidant activity [86].

### 3.7. Sensory Properties

The sensory analysis of the treated samples is essential, since it indicates the acceptability on the part of the final consumer of the fruits conserved with the coatings [52].

**Figure 13:**
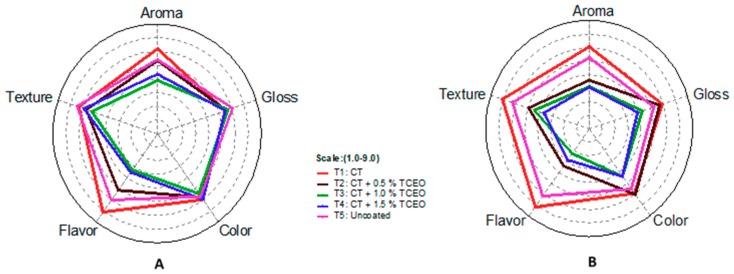
A hedonistic scale of strawberries with CT-TCEO on day 4 (**A**) and day 7(**B).**

The results of the hedonic evaluations are observed in Figure 13A during the four days of storage. Results show that the fruits with T2 had higher acceptance regarding aroma attributes and flavor, with a significant difference (*p* < 0.05); however, the analysis of texture perception and color was higher in T4 but did not present a significant difference when compared to the treatments that have essential oil. Regarding day 7 of storage (Figure 13B), fruits with T2 had a greater acceptance of the sensory attributes evaluated against the treatments with essential oil.

The attributes that had lower acceptability for the sensory panel on day 4 were in the fruits with T3 applied, for most of the sensory attributes, while on day 7, the treatment with low acceptability was T4, probably due to the higher bitter taste and the lack of sensory attributes suitable for the panel. This behavior indicates that the treatments with higher concentrations of essential oil incorporated in chitosan change the original organoleptic characteristics of the strawberries; accordingly, it is important in future studies to adjust the way to mask the flavor with the application of other substances that are of low commercial value but that can facilitate its acceptance. As it has been observed before by [87], mechanical strength decreased with thyme oil incorporation, which means that the perception of texture also should change by the incorporation of the TCEO.

It was determined that the treatment with greater acceptability in the applied sensory tests was T2 and the one with low acceptability was T4.

## 4. Conclusions

The results of the present study showed that the incorporation of *Thymus capitatus* essential oil in edible fruit coatings made of chitosan increased the shelf-life of the strawberries for at least 15 days under 5 °C.

The fruits exhibited excellent stability with respect to fungal decay, loss of moisture, maturation, respiration rate, and conservation of antioxidant activity, among other characteristics.

The treatments CT-TCEO positively impacted the physicochemical composition of the fruit, so that they comply with the attributes established by NTC 4103. Besides, they can delay the microbial development of aerobic mesophylls, molds, and yeasts, in comparison with samples without treatment or treated only with chitosan by one logarithmic unit. All these observations demonstrated a positive effect in decreasing the microbial population and extending the shelf-life of strawberries. The treatment with the highest content of TCEO (T4) was the most effective; however, at a sensory level, the coating did not have acceptance due to the strange flavors that conferred to strawberry fruits, implicating future investigation to encapsulate or mask the taste of oil.

The analysis of the physicochemical properties of the emulsions, such as apparent viscosity and particle size, indicated that the methodology used allowed for the obtaining of microemulsions without problems of separation and easy application to the strawberries, as a result of a low dispersion in particle size.

The morphological analysis of the film did not show significant differences between the oil concentrations, while the cross-section showed the incorporation of the oil between the chitosan matrix, and the presence of a porous structure and an increasing of roughness, without affecting their stability, since they are dispersed between the chitosan chains by the action of the surfactant used and the high speed of agitation.

In the present investigation, an alternative method has been proposed to prolong the shelf-life of the strawberries, with applications in the food industry; however, TCEO must be incorporated at a lower concentration in the coatings in order to minimize its impact on sensory perception, achieving greater acceptance in the organoleptic parameters evaluated, or using a masking agent of the flavor.

## Figures and Tables

**Table 1:** Volatile compounds expressed as area percentage, identified in the *Thymus capitatus* essential oil [58].

	Compound	RT	Amount Relative (%)	*KI
Monoterpenes hydrocarbons	Tricyclene	17.15	<0.1	920
	α-Thujene	17.26	0.1	923
	α-Pinene	17.65	1.5	935
	α-Fenchene	18.43	0.3	951
	β-Pinene	19.67	0.3	981
	β-Myrcene	20.03	2.0	991
	*p*-Mentha-1(7),8-diene	20.78	0.1	992
	α-Phellandrene	20.88	0.2	1005
	δ−3−Carene	20.99	<0.1	1012
	1,4-Cineole	21.23	<0.1	1014
	α-Terpinene	21.34	1.5	1018
	*p*-Cymene	21.74	13.2	1026
	Limonene	21.89	0.4	1033
	1,8-cineole	22.05	0.4	1033
	γ-Terpinene	23.13	8.7	1064
	N.I. (M + 154)	23.63	0.1	
Monoterpenes oxygenated	Terpinolene	24.24	0.2	1078
	Linalool	24.73	1.9	1100
	Borneol	27.87	0.3	1165
	Terpinen-4-ol	28.15	0.6	1190
	α-Terpineol	28.79	0.1	1200
	Thymol	32.07	6.4	1266
	Carvacrol	32.63	59.3	1278
	*trans*-β-Caryophyllene	37.34	2.2	1424
Sesquiterpenes oxygenated	Caryophyllene oxide	42.50	0.2	1581

*KI is the Kováts Retention Index relative to C5–C24 n-alkanes on the DB-5 column.

**Table 2:** Physical properties of the CT-TCEO coatings.

Essential Oil (%)	Gardner Viscosity * (Scale)	Apparent Viscosity (cP)	Solids (%)	Particle Size (μm)
0.5	E	125	2.70 ± 0.01	1.23 ± 0.25
1.0	C	85	2.76 ± 0.02	1.14 ± 0.32
1.5	B	65	3.27 ± 0.02	1.13 ± 0.12

* The range reported is the result of more than three repetitions, and because the result is reported in the letter, there was no difference in the values obtained.
